# Improved RRT* Algorithm for Disinfecting Robot Path Planning

**DOI:** 10.3390/s24051520

**Published:** 2024-02-26

**Authors:** Haotian Wang, Xiaolong Zhou, Jianyong Li, Zhilun Yang, Linlin Cao

**Affiliations:** Mechanical Engineering College, Beihua University, Jilin 132021, China; wanghaotian0103@163.com (H.W.);

**Keywords:** disinfection robot, artificial potential field (APF), fuzzy control, rapidly exploring random trees (RRT*), path planning

## Abstract

In this paper, an improved APF-GFARRT* (artificial potential field-guided fuzzy adaptive rapidly exploring random trees) algorithm based on APF (artificial potential field) guided sampling and fuzzy adaptive expansion is proposed to solve the problems of weak orientation and low search success rate when randomly expanding nodes using the RRT (rapidly exploring random trees) algorithm for disinfecting robots in the dense environment of disinfection operation. Considering the inherent randomness of tree growth in the RRT* algorithm, a combination of APF with RRT* is introduced to enhance the purposefulness of the sampling process. In addition, in the context of RRT* facing dense and restricted environments such as narrow passages, adaptive step-size adjustment is implemented using fuzzy control. It accelerates the algorithm’s convergence and improves search efficiency in a specific area. The proposed algorithm is validated and analyzed in a specialized environment designed in MATLAB, and comparisons are made with existing path planning algorithms, including RRT, RRT*, and APF-RRT*. Experimental results show the excellent exploration speed of the improved algorithm, reducing the average initial path search time by about 46.52% compared to the other three algorithms. In addition, the improved algorithm exhibits faster convergence, significantly reducing the average iteration count and the average final path cost by about 10.01%. The algorithm’s enhanced adaptability in unique environments is particularly noteworthy, increasing the chances of successfully finding paths and generating more rational and smoother paths than other algorithms. Experimental results validate the proposed algorithm as a practical and feasible solution for similar problems.

## 1. Introduction

In recent years, the continuous advancement of robotic technology has found widespread applications in various fields. During the global pandemic, robots have taken on tasks such as temperature measurement, drug delivery, and disinfection, effectively alleviating the pressure of workforce shortages and reducing the risk of cross-infections [1,2]. The related technologies mainly involve real-time localization, environment sensing, path planning, and autonomous control technologies, among which path planning and autonomous obstacle avoidance have been the focus of research [3,4]. The path-planning technology of the robot collects external information mainly through sensors such as radars and visual cameras, and the controller of the robot can build reliable travel routes [5,6,7]. However, in practical disinfection tasks, the effectiveness of path planning is affected by various factors [8]. For example, when there are obstacles around the target point, it will increase the computational load, leading to less efficient execution of the algorithm. Similarly, existing algorithms may have robots stalling or wandering when they need to pass through a narrow passage and are unable to plan paths efficiently. The above two special cases make the existing disinfection robots not fully effective in some specific environments. Hence, it is crucial to enhance the performance of disinfection robots with the optimization and improvement of path-planning algorithms.

Currently, the path planning algorithms based on grid search (Dijkstra, A*), the path planning algorithm based on virtual potential field (APF), the path planning algorithms based on probabilistic sampling (probabilistic roadmap method, RRT), and the path planning algorithms based on bionic intelligence (simulated annealing, genetic algorithm, particle swarm optimization) are the widely used ones for mobile robots. Among them, the A* algorithm has a strong search capability but is affected by mesh modeling in complex environments [9,10,11]. The APF algorithm has a simple structure with high real-time performance, but path oscillations may occur in dense obstacle regions [12,13,14,15,16]. The simulated annealing algorithm can get rid of the local optimum and converge to the global optimum solution, but its convergence is relatively slow due to the influence of the temperature cooling rate [17,18]. Genetic algorithms are easily combined with other optimization algorithms or heuristics to improve performance but have a high computational complexity [19,20]. The PSO algorithm has the characteristics of memory and fast convergence, but it also suffers from obvious shortcomings such as poor detection ability, insufficient particle diversity, and susceptibility to local minima [21,22,23]. Accordingly, the RRT algorithm, employing a globally uniform random sampling strategy for rapidly expanding new nodes, stands out as an optimal choice for scenarios requiring rapid path planning [24,25,26,27]. However, as task complexity increases, researchers have identified some limitations in RRT, such as low node utilization [28,29] and path instability [30,31].

Several scholars have conducted in-depth studies to deal with these shortcomings, proposing effective methods to enhance RRT algorithms. Incorporating the bidirectional search concept, Kuffner introduced the RRT-Connect algorithm [32]. This algorithm uses heuristic steps to reduce futile searches in blank areas, ultimately saving overall search time. Building upon this, Karaman proposed an enhanced RRT algorithm [33]. RRT* contains a ‘Rewire’ process during tree growth, optimizing tree structure through parent node reselection and pruning operations. After several iterations, RRT* converges to an asymptotically optimal solution. Gammell et al. proposed the Informed-RRT* algorithm based on RRT* [34]. This algorithm decreases the search for unnecessary areas by limiting the sampling space to an elliptical region and gradually decreasing the sampling area as the path length decreases. The cost of the route is lower than RRT*.

While numerous researchers have been quite effective in improving the sampling and expansion methods and limiting the sampling domain of RRT algorithms, these improved algorithms still cannot be directly applied to disinfecting robot systems. For example, the RRT-Connect algorithm is not sufficiently goal-directed when constructing a two-way tree, which might lead to more random paths despite fast convergence. RRT* introduces a “rewire” process to optimize the structure of the search tree, but in dense obstacles, which have narrow passages and entrances, the reconnection process is limited to converge, which prolongs the time to obtain the global optimal solution. Furthermore, although the Informed-RRT* algorithm improves the search efficiency by limiting the sampling range, the performance of the algorithm depends mainly on the setting of ellipsoidal region parameters, which need to be continuously adjusted in different scenarios to achieve the best results, which increases the complexity of the practical application to a certain extent. In order to solve the above problems, this paper proposes an improved RRT* algorithm based on APF guided sampling and combined with fuzzy adaptive extension, aiming to solve the path planning problem of disinfecting robots better.

The remaining structure of this paper is as follows: Section 2 provides a detailed exposition of the proposed method and relevant knowledge. Additionally, Section 3 presents a comprehensive overview of the experimental design and implementation results, validating the performance and advantages of the proposed method. Finally, Section 4 discusses the experimental results and concludes with a summary of the research question. 

## 2. Method

### 2.1. Basic RRT* Algorithm

The basic RRT search process resembles the growth of a tree expanding in all directions, with the initial node $X_{init}$ representing the root of the tree. The random function generates a random node $X_{rand}$ within the free space. Using the nearest function, it calculates Euclidean distance and selects the node $X_{near}$, which is closest to $X_{rand}$.

**Figure 1:**
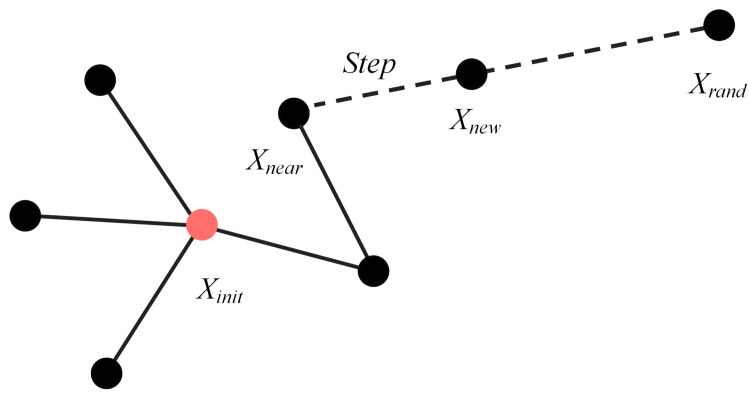
Schematic diagram of RRT expansion.

Subsequently, along the direction from $X_{near}$ to $X_{rand}$, the expansion occurs with Step. The algorithm uses the resulting new $X_{new}$ node for collision detection. If the line segment between $X_{new}$ and $X_{near}$ is collision-free, the algorithm adds $X_{new}$ to the tree T as a child node. Otherwise, the algorithm discards $X_{new}$, and the current iteration ends, moving on to the next iteration. Figure 1 illustrates the process of expanding a new node.

**Figure 2:**
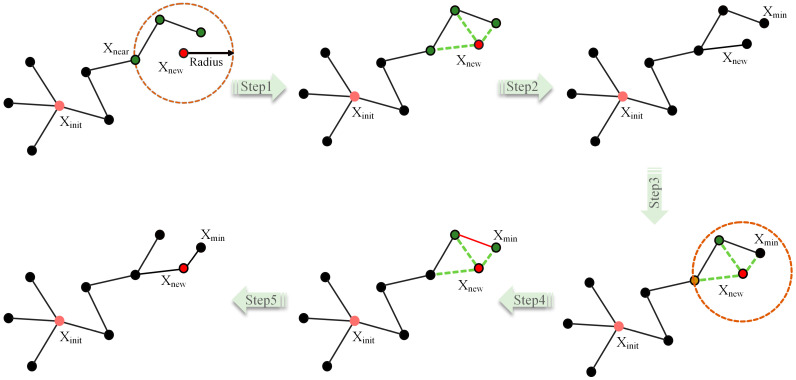
Schematic diagram of RRT* algorithm expansion.

The RRT* algorithm enhances the basic RRT algorithm by introducing an accumulated cost attribute, which records the sum of the lengths of all edges along the path from the starting point to the respective node. A new strategy is implemented that involves the re-selection of the parent node for $X_{new}$ instead of using $X_{near}$ as the parent node. The selection process evaluates all nodes within a certain radius, with $X_{new}$ as the center, and selects the node with the lowest cost as the best parent node for $X_{new}$. Once the optimal parent node is determined, the algorithm traverses the remaining tree nodes, calculating the path cost from each node to both $X_{new}$ and root node $X_{init}$. The algorithm reconstructs the tree by choosing the path with the minimum accumulated cost. Figure 2 illustrates the entire process of optimizing the parent node and reconstructing the backtracking.

### 2.2. Implementation of the Improved Algorithm

The RRT* algorithm undergoes the two key processing steps described above, significantly improving the selection of nearby nodes and path optimization compared to the RRT algorithm. However, it performs consistently with RRT in areas with narrow passages and entrances and is prone to local growth difficulties. Aiming at the low exploration efficiency of the RRT* algorithm in particular areas and the redundant nodes that still exist in the search path, this paper proposes the improved APF-GFARRT* (artificial potential field-guided fuzzy adaptive rapidly exploring random trees) algorithm. The algorithm consists of two key modules: the sampling point guidance module and the adaptive step-size adjustment module. The sampling point guidance module uses the APF and sets the target point as a potential field attractive point to attract sampling points. In addition, in order to improve the speed of the algorithm searching for the first executable path, the adaptive step-size adjustment module is introduced, which applies fuzzy control for adaptive step-size adjustment to accelerate the convergence speed of the algorithm. Finally, the fuzzy controller introduces the contract expansion factor to adaptively adjust the step-size output outside the target area to decrease the exploration of unnecessary areas.

#### 2.2.1. Sampling Point Guidance Module

RRT expands new nodes with a globally uniform random sampling strategy to generate a feasible path quickly. However, the random nature of the algorithm can lead to path search becoming blind and inefficient in dense environments. Therefore, introducing a sampling point guidance module is particularly important to improve the algorithm’s guidance in narrow passages and entrances.

Khatib first proposed the classical APF [16]. The core idea of this method is to model the robot’s surroundings as a virtual potential field in which the target point generates a continuous attraction force, and obstacles generate a repulsive force. Planning a route using the attraction force of the target point and the repulsive force of the obstacles is expressed as follows:

The total potential field U is expressed as a superposition of the attractive field function $U_{att}$ and the repulsive field function $U_{req}$:

(1) $$U(x)=U_{att}(x)+U_{req}(x)$$

The following equation expresses the attractive field function $U_{att}$:

(2) $$U_{att}(X)=\frac{1}{2}K_{att}(X-X_{target}{)}^{2}$$

where $K_{att}$ is the attractive factor, which regulates the magnitude of the potential energy of the attractive field, and the target point coordinates are $X_{target}$. The following equation then describes the repulsive field function $U_{req}(x)$:

(3) $$U_{rep}(X)=\left\{\begin{array}{r} \frac{1}{2}\eta {\left(\frac{1}{\rho \left(X,X_{obs}\right)}-\frac{1}{p_{0}}\right)}^{2}\left(X-X_{target}\right),\rho \left(X,X_{obs}\right)<p_{0}\\ 0,\rho \left(X,X_{obs}\right)\geq p_{0} \end{array}\right.$$

where $\rho \left(X,X_{obs}\right)$ denotes the shortest distance between the current position of the robot and the position of the obstacle, η is the position gain function, $p_{0}$ is the maximum distance at which the obstacle acts as a repulsive force on the robot, and the repulsive force will be 0 when it exceeds the distance of the action. Suppose the number of practical obstacles on the search path is m. The APF generates a superimposed force on the robot, expressed as:

(4) $$F(X)=F_{att}(X)+\sum_{i=1}^{m}F_{rep}(X)$$

The APF has the advantages of a simple structure and fast information processing speed. In order to improve the goal orientation of node expansion, the APF is introduced in the RRT* expansion process. Considering that the APF is prone to local constraints in the path search process, the potential field strength decreases to 0. Therefore, only the effect of the attractive field is retained in the search process to avoid APF-GFARRT* falling into the local minimum point.

After obtaining the coordinate information of the random point $X_{rand}$, the sampling point guidance module will set the target point $X_{target}$ as the attractive point. The sampling point guidance module calculates the attraction level and direction received by $X_{rand}$ to generate the corresponding guide point, and the guide point will replace the random point. The process is as follows:

Set the coordinates of the current random point $X_{rand}=(x_{rand},y_{rand})$, then the displacement vector of the target point relative to the random point is $\vec{v}=(v_{x},v_{y})$, where:

(5) $$v_{x}=x_{target}-x_{rand}$$

(6) $$v_{y}=y_{target}-y_{rand}$$

Then the angle vector θ can be calculated using the inverse tangent function:

(7) $$\theta =actan2(v_{x},v_{y})$$

Suppose the random point does not overlap with the target point. In that case, the potential attraction force function is inversely proportional to the distance from the above Equation (2), so the attraction force function $F_{att}(X)$:

(8) $$F_{att}(X)=-\nabla U_{att}(X)=-K_{att}(X-X_{target})$$

Suppose the Euclidean distance between two points is d and attractive factor is $K_{attr}$. Then the attraction force $F_{attr}$ on the random point is:

(9) $$F_{attr}=K_{attr}\cdot d$$

The distance d is:

(10) $$d=\sqrt{{\left(x_{target}-x_{rand}\right)}^{2}+{\left(y_{target}-y_{rand}\right)}^{2}}$$

By combining (6)–(9), we can calculate the components of the attraction force $F_{attr}$ amid the Cartesian coordinate system:

(11) $$F_{attr,x}=F_{attr}\cdot \cos\theta$$

(12) $$F_{attr,y}=F_{attr}\cdot \sin\theta$$

The coordinates of the final guiding point $x_{guided}$ can be calculated using the following equation:

(13) $$x_{guided}=x_{rand}+\xi \cdot F_{attr,x}$$

(14) $$y_{guided}=y_{rand}+\xi \cdot F_{attr,y}$$

Here, ξ is the guiding coefficient, which controls the generated position of the guiding point.

**Figure 3:**
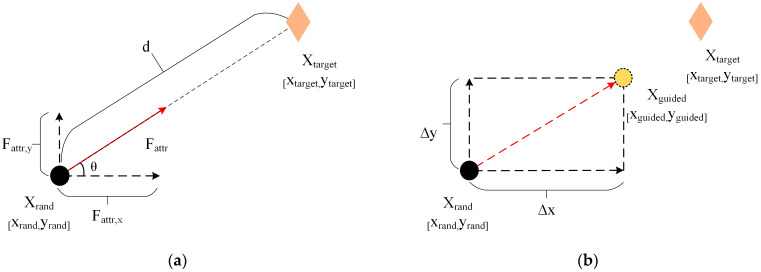
(**a**) Calculate the gravitational force. (**b**) Generate the guiding point.

After setting up the attractive field, the process of generating bootstrap points is shown in Figure 3. Although sampling points still have a certain degree of randomness, the random tree will be more inclined to grow in the direction of the target point under the influence of the potential field.

#### 2.2.2. Adaptive Step-Size Adjustment Module

In RRT*, the tree growth length is determined by the parameter step size, too large or too small will affect the search performance. In order to balance the depth and breadth of the path search process, this subsection introduces fuzzy control to adaptively adjust the step size.

**Figure 4:**
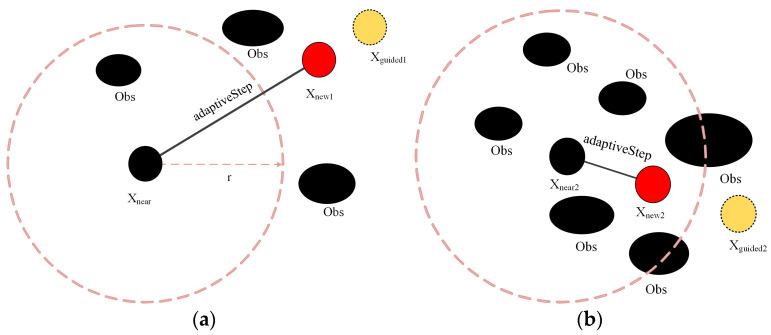
Adaptive step-size adjustment: (**a**) open areas; (**b**) narrow.

There are two main points to consider when designing a fuzzy controller: the density of obstacles in the neighborhood of the sampling point (ObsDensity) and the distance from the current node to the target point (PointDist). Firstly, the obstacle density is used as one of the input variables to evaluate environmental complexity. For areas with dense environments, the step size should be reduced accordingly for accurate search in small spaces. On the contrary, in areas with fewer obstacles, the step size can be increased, which improves search efficiency. Secondly, the distance from the current point to the target point is taken as another control parameter, and the step size is adjusted according to the distance size. The schematic diagram of the adaptive step length adjustment is shown in Figure 4 below.

**Figure 5:**
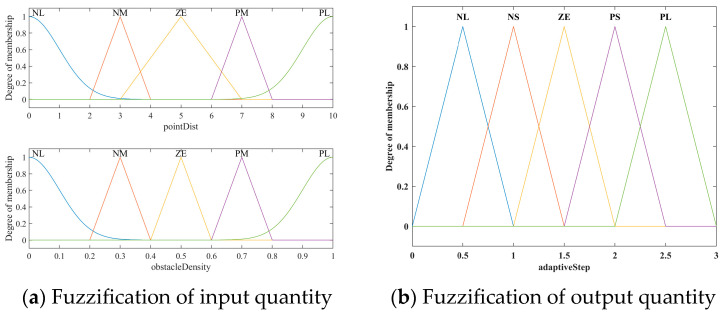
Membership function setting for inputs and output.

In the fuzzy system, ObsDensity and PointDist inputs are configured as identical fuzzy subsets {NL, NM, ZE, PM, PL}. After normalization, their value ranges are (0, 1) and (0, 10), respectively. In addition, five membership function (MF) statuses, PL, PS, ZE, NS, and NL, are assigned for the AdaptiveStep output. Here, ‘L’, ‘M’, and ‘S’ represent ‘Large’, ‘Medium’, and ‘Small’, respectively. The MF adopts symmetrical, uniformly distributed triangular and Gaussian shapes. The shapes of the input and output MF are illustrated in Figure 5. Subsequently, twenty-five fuzzy rules are designed, as shown in Table 1. Mamdani and Centroid methods are employed for fuzzy inference and defuzzification in the fuzzy system, enhancing its adaptive adjustment performance.

During the RRT* iteration, even if the first feasible path is successfully found, the global uniform sampling strategy remains unchanged, which may lead to repeated sampling in the low-value area and make it difficult to converge quickly. To solve this problem, a scaling factor α is designed which effectively adjusts the output range of the fuzzy controller. The fuzzy subset of this factor contains {VB, B, M, S, V, VS} and sets the output domain to be only related to its variation. The α expression is as follows:

(15) $$\alpha =e^{-k_{1}\cdot D_{line}}$$

Here, $k_{1}$ is a proportional constant, and $D_{line}$ represents the vertical distance from the sampling point to the line L (the line defined by the starting and target point). The expression is given by:

(16) $$D_{line}=\frac{\left|(y_{guided}-y_{star})(x_{target}-x_{star})-\left.\left(x_{guided}-x_{star})(y_{target}-y_{star}\right)\right|\right.}{\sqrt{{\left(x_{target}-x_{star}\right)}^{2}+{\left(y_{target}-y_{star}\right)}^{2}}}$$

The role of α is to accelerate the convergence of the algorithm. Specifically, it determines whether a sampling point is located in a low-value region by calculating the $D_{line}$. If $D_{line}$ exceeds a set threshold, α will change the output range of the fuzzy controller, thus affecting the rapid decrease in adaptiveStep as the distance from $D_{line}$ increases, and effectively preventing the random tree from exploring the low-value region during the reconstruction process.

**Figure 6:**
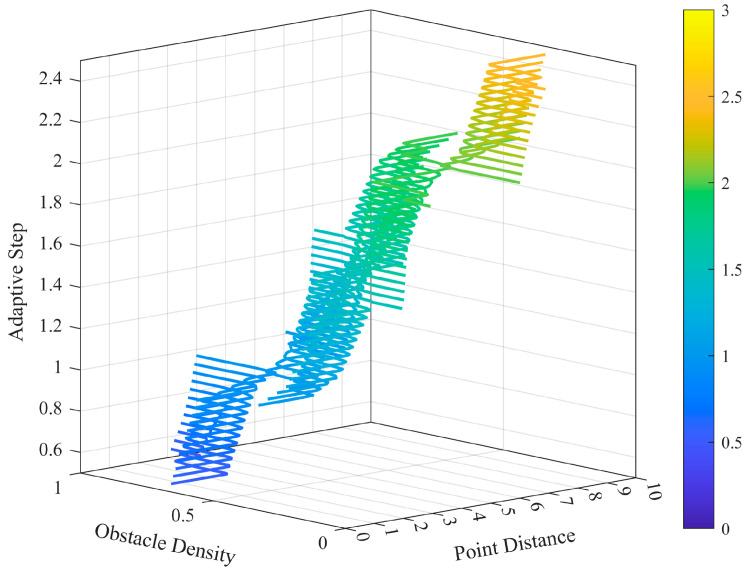
Adaptive step variation illustration in fuzzy control systems.

Up to this point, the primary controller design is completed, and the variation of the adaptive step under different input conditions is illustrated in Figure 6.

#### 2.2.3. Implementation of the Improved Algorithm

**Figure 7:**
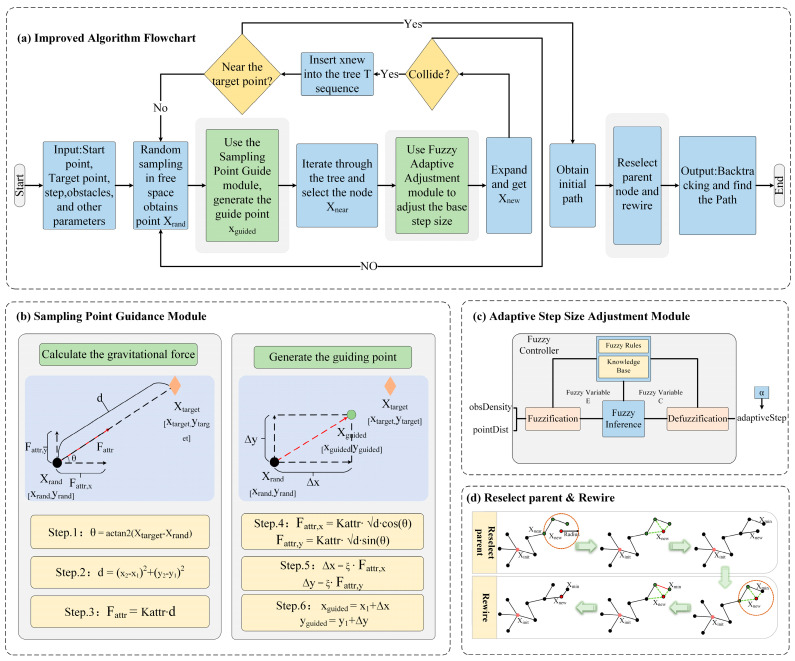
Flow chart of APF-GFARRT* path-planning algorithm based on adaptive step size.

By combining the sampling point guidance module and the adaptive step-size adjustment module, this paper obtain the improved APF-GFARRT* algorithm based on guidance sampling and fuzzy adaptive extension. The overall flow of the path planning algorithm is shown in Figure 7.

The pseudocode (Algorithm 1) describes the process of APF-GFARRT* searching for the path, and its main steps are as follows:

1. Initialization (lines 1–2): Initiate the whole program, set the start point $X_{init}$, the target point $X_{target}$, and other parameters, and create an empty tree T with $X_{init}$ as the root node.
2. Main loop (iterative loop, lines 3–28):
  ➢ Lines 4–6: Generate a random point $X_{rand}$, calculate the attraction force apf_force, and get the coordinates of the guidance point.
  ➢ Lines 7–8: Calculate $D_{line}$ and search for node $X_{near}$ in tree T.
  ➢ Lines 9–24: Call CollisionFree function to check if the line from $X_{near}$ to $x_{guided}$ crosses the obstacle.
  ➢ Line 10–11: Call the function to calculate the obstacle density level at the guided point and output adaptiveStep according to the fuzzy rules.
  ➢ Line 12–17: Check whether the first feasible path has been searched, if findPath is 1, then it is currently in the path optimization stage, adjust the extended step size according to the scaling factor α. Next, expand the $X_{new}$.
  ➢ Lines 18–23: Select the parent node and update the tree structure.
  ➢ Lines 25–27: Check if the new node reaches the target point or nearby area, if yes, add the target point into the tree structure and backtrack the final path.
3. Return result (line 29): If the maximum number of iterations is reached and no path is found, then return no path.

**Algorithm 1** ${\mathrm{APF-GFARRT}}^{*}$ Algorithm01: Initialize $X_{init}$, $X_{goal}$, and other parameters.02: Initialize an empty tree T with the root node as $X_{init}$03: **for** i=1 to MaxIterations **do**04:  $X_{rand}\leftarrow \mathrm{NewRandPoint}\left(\right)$05:  $\mathrm{apf\_force}\leftarrow \mathrm{APF\_att}\left(X_{rand},X_{goal}\right)$06:  $X_{guided}\leftarrow \mathrm{GenerateGuided}\left(X_{rand},\mathrm{apf\_force}\right)$07:  $D_{line}\leftarrow \mathrm{Distance}\left(X_{guided},X_{start},X_{goal}\right)$08:  $X_{near}\leftarrow \mathrm{Nearest}\left(T,X_{guided}\right)$09:  **If** $\mathrm{CollisionFree}\left(X_{near},\;X_{guided}\right)$ **then**10:   $obsDensity\leftarrow \mathrm{ObstacleDensity}\left(X_{guided},\;\mathrm{fixedRadius}\right)$11:   $adaptiveStep\leftarrow \mathrm{FuzzyAdaptiveStep}{\left(obsDensity\right)}^{*}Step$12:   **If** findPath==0 **then**13:    $X_{new}\leftarrow \mathrm{Steer}\left(X_{near},X_{guided},adaptiveStep\right)$14:   **else**15:    $\alpha \leftarrow \left(D_{line}\geq D_{Threshold}\right)?\;\mathrm{ComputeAlpha}\left(K,D_{line}\right):1$16:    $X_{new}\leftarrow \mathrm{Steer}\left(X_{near},X_{guided},adaptiveStep*\alpha \right)$17:   **end if**18:   $X_{nearest}\leftarrow \mathrm{NearC}\left(T,X_{new},r\right)$19:   $X_{min}\leftarrow \mathrm{ChooseBestParent}(X_{nearest},X_{new})$20:   $\mathrm{AddNodeEdge}(T,X_{min})$21:   $X_{near}^{newparent}\leftarrow \mathrm{UpdateParent}(X_{new},X_{nearest})$22:   $\mathrm{PruneNodes}\left(X_{near}^{newparent},X_{nearest}\right)$23:   $\mathrm{Rewire}\left(T\right)$24:  **end if**25:  **if** $\mathrm{GoalReached}\left(X_{new},\;X_{guided}\right)$ **then**26:   **return** $\mathrm{ReconstructPath}\left(T,X_{init},X_{goal}\right)$27:  **end if**28: **end for**29: **return** $\mathrm{NoPathFound}\left(\right)$

## 3. Experiment and Analysis

In this section, the APF-GFARRT* algorithm will be experimentally validated by comparing it with the existing RRT, RRT*, and APF-RRT* [35] algorithms in the same 2D environment to verify the performance of the APF-GFDARRT* algorithm. The simulation platform and configuration include MATLAB R2022a, 64-bit Windows 10, and AMD Ryzen3 5600 processor with a CPU frequency of 3.6 GHz and 8 GB RAM.

**Figure 8:**
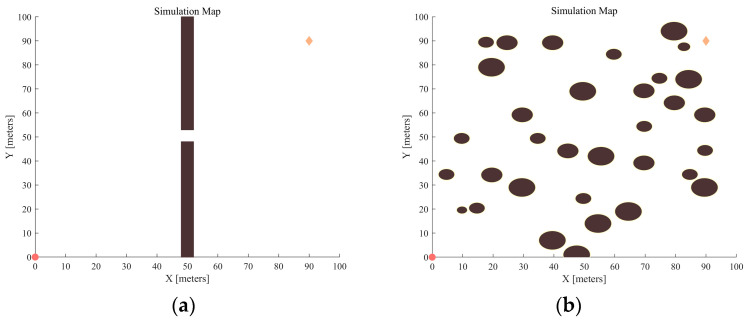
Two different environmental map models: (**a**) Map 1; (**b**) Map 2. In these maps, the starting point is represented by a circle, and the end point is represented by a diamond.

During the experiments, the maximum number of iterations is set to 1000, the attractive factor is 50, and the step size is fixed at 2.5 M. In the APF-GFARRT* algorithm, a crucial adjustment is made by setting the minimum step size to 1 M to avoid unnecessary redundant nodes resulting from the small step size. To maintain consistency and comparability, other relevant parameters are kept unchanged in the experiments. Additionally, the map size for all experimental environments is standardized at [100, 100], with the start and end points defined as [0, 0] and [90, 90], respectively. Considering the collision volume of the disinfecting robot, the robot is simplified to a material point, and the obstacles are moderately inflated. Figure 8 shows the distribution of obstacles and start and end nodes of both maps.

### 3.1. Narrow Passage Testing

**Figure 9:**
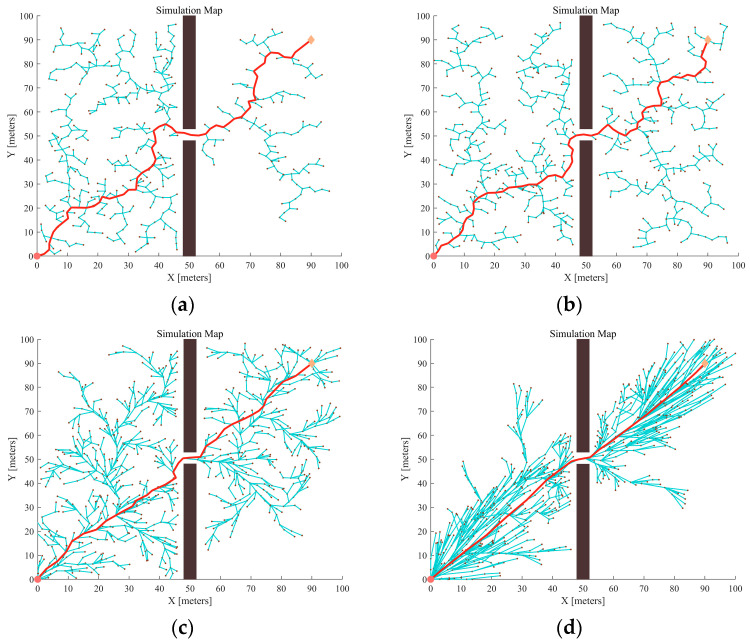
Different algorithms generate the path in the same map (Map 1): (**a**) RRT; (**b**) RRT*; (**c**) APF-RRT*; (**d**) APF-GFARRT*.

The RRT and RRT* algorithms may be limited in narrow passages due to the lack of effective bootstrapping, and their expansion may be stagnant or significantly slow. To verify the reliability of algorithms such as APF-GFRRT* in restricted environments, a narrow passage was set up in Map 1, and 100 sets of experiments were conducted. The experimental results are shown in Figure 9 and the experimental data are summarized in Table 2.

In Figure 9, the red curve represents the final path, and the blue lines represent the individual branch points derived during the random tree expansion process. Comparing the four planning results in Figure 9, it can be concluded that APF-GFARRT* performs best in finding paths to narrow areas. Furthermore, Figure 9d demonstrates that the improved algorithm has a denser distribution of sampling points near the narrow passage. This is because the addition of the sampling guidance module has a stronger orientation and the random tree tends to grow more toward the target point.

**Figure 10:**
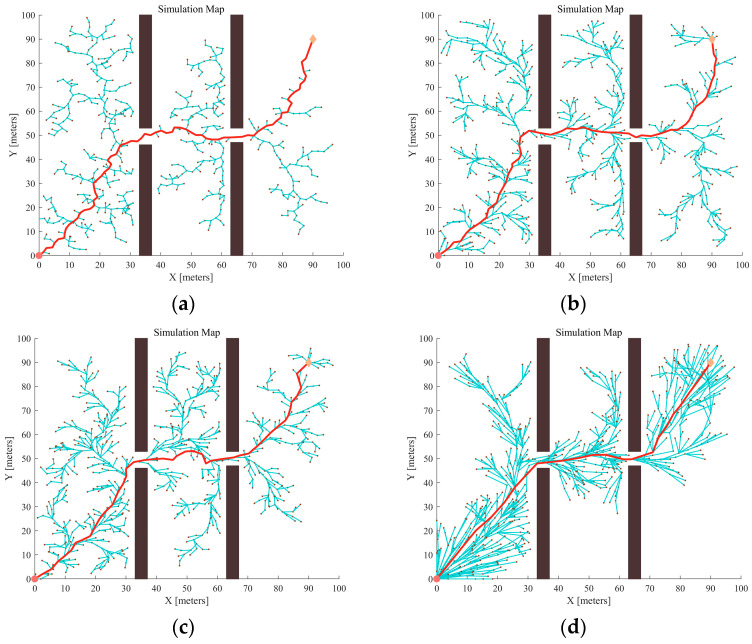
Different algorithms generate the path in the same obstacle environment: (**a**) RRT; (**b**) RRT*; (**c**) APF-RRT*; (**d**) APF-GFARRT*.

To validate the effectiveness of the proposed algorithm in overcoming narrow-area constraints, two restricted passages were set up in the Map 1 environment, and four algorithms were used for planning. The results are shown in Figure 10:

Compared with Figure 9, Figure 10 shows that when adding constrained areas in the map, the growth rate of the random tree is significantly reduced, and creates more forks. Furthermore, it can be derived from the data in Table 3 that the success probabilities of the RRT and RRT* algorithms also decrease. Meanwhile, the average path cost also increases. In contrast, after the addition of artificial potential field guidance, the sampling points of APF-RRT* and APF-GFRRT* are more inclined to the target point, but as can be seen in Figure 10c, APF-RRT* still stagnates at narrow passages and the success rate decreases. The success rate of APF-GFRRT* with fuzzy adaptive step-size adjustment strategy stays above 85%. In addition, the random tree is more directional and faster in the process of expanding to the target point, as shown in Figure 10d.

**Figure 11:**
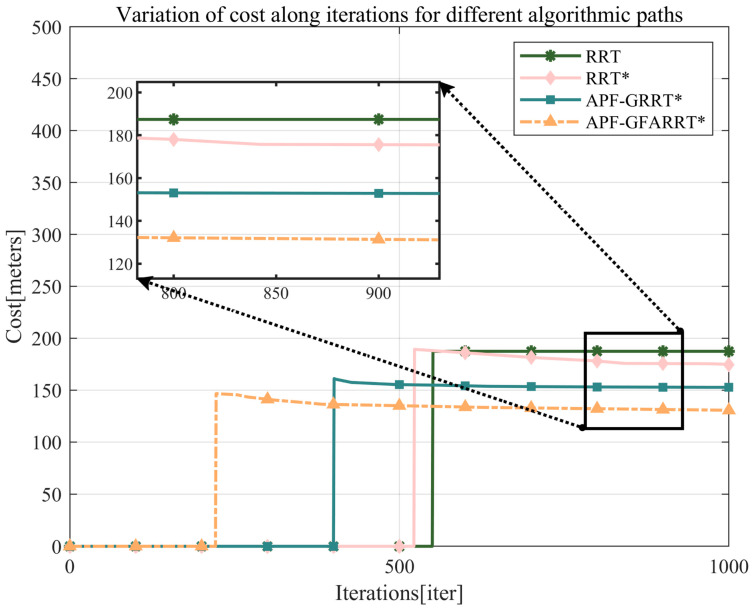
Path length convergence curves for APF-GFRRT* and other algorithms.

From the convergence curves of the path lengths of the APF-GFRRT* algorithm and the traditional algorithm in Figure 11, it can be seen that the improved algorithm still has a shorter path length and converges to the asymptotically optimal path with fewer iterations, which verifies the reliability of the algorithm.

### 3.2. Dense Obstacle Testing

Dense obstacles were set up in Map 2 to verify the APF-GFARRT* algorithm’s reliability in dense environments. There were two hundred experiments conducted for each algorithm to record data, for instance, the time taken to find the path for the first time, the average number of path nodes, and the average path cost during the experiments. Table 4 provides a comparative analysis of these data for various algorithms.

**Figure 12:**
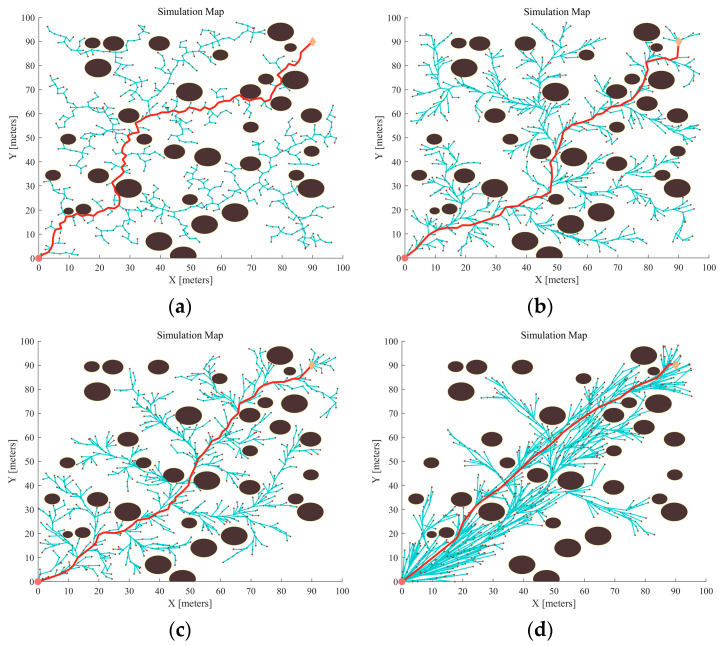
Different algorithms generate the path in the same map (Map 2): (**a**) RRT; (**b**) RRT*; (**c**) APF-RRT*; (**d**) APF-GFARRT*.

Figure 12a shows the RRT algorithm’s path results. The RRT* algorithm planning results are shown in Figure 12b. The outcomes of the iterative experiment of the APF-RRT* algorithm are shown in Figure 12c, and the improved planning effect of the APF-GFARRT* algorithm is shown in Figure 12d. By observing Figure 12d, it can be found that the improved APF-GFARRT* algorithm adopts a larger step size for expansion in the area with fewer obstacles in the lower left corner and more open terrain, while in the area with dense obstacles in the center of the map, the step size is adjusted and reduced accordingly (the program sets the minimum step size to 1 m to prevent expansion caused by too small a step size). Compared with other algorithms, the improved algorithm has improved adaptive capability after introducing the adaptive adjustment module, and the number of path nodes has been significantly decreased. Comparing Figure 12c with Figure 12d, the APF-GFARRT* algorithm limits the expansion rate beyond the specified sampling area by contracting expanding factors. The algorithm focuses on sampling optimization in the region near the initial path, leading to a smoother final path and requiring a smaller path cost. Comparing the planning results of other algorithms in Figure 12, it can be found that the APF-GFARRT* algorithm has a better final planning effect with the same number of iterations.

According to the data in Table 4, it can be concluded that both RRT and RRT* have a probability of failure during the search process, with success rates of 73% and 84%, respectively. The reason is that both algorithms use global random sampling, which brings large-scale sampling costs to find feasible paths in complex environments. In addition, the dense obstacles create many narrow areas, which seriously affect the search rate of RRT and RRT* algorithms, and the search times for the first path are 6.8701 s and 6.6804 s, respectively.

After comparative analysis, the average path cost and the average number of path nodes of the original RRT algorithm are 160.6656 and 81.1864, respectively, but with the addition of the ‘rewire’ mechanism, these two values are decreased to 142.4439 and 46.3722, respectively. The APF-RRT* algorithm with the help of an artificial potential field-oriented strategy decreases the average path length and the average number of path nodes to 138.9890 and 45.8793, which further improves the search performance. Compared with the traditional RRT, RRT*, and APF-RRT* algorithms, the APF-GFARRT* algorithm spends the shortest time to find feasible paths, which is only 3.5721 s. In addition, benefitting from the artificial potential field sampling guidance, the APF-GFARRT* algorithm has more outstanding search rate in dense environments, and it is equipped with adaptive step-size adjustment, which ultimately decreases the average path cost and the average number of path nodes to a lower level: 128.35 and 29.2977.

**Figure 13:**
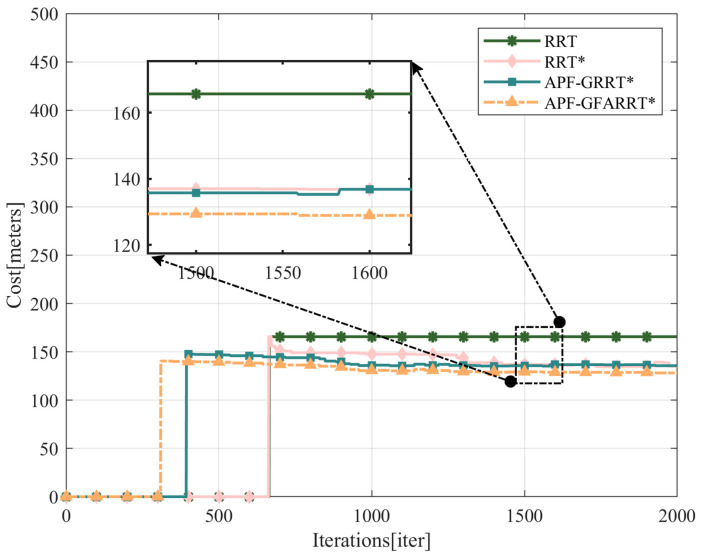
Variation of cost along iterations for different algorithmic paths.

The RRT*, APF-RRT*, and APF-GFARRT* algorithms are all asymptotically optimal, so there is no significant difference in their average path costs in the same iterations. However, the APF-GFARRT* algorithm performs faster in finding the first feasible path. As can be seen in Figure 13, although there is no significant difference in cost at the later stage of the path, the path length of the APF-GFARRT* algorithm after 500 iterations is much smaller than that of the RRT* algorithm after 1500 iterations, which is closer to the optimal path. It is verified that the proposed sampling guide module and adaptive step-size adjustment module effectively improve the algorithm’s convergence rate so that the path closer to the optimal solution can be found with fewer iterations.

## 4. Conclusions and Future Work

Currently, in the face of the severe challenges of global public health problems, disinfection robots, as an epidemic prevention method, are gradually widely used in major public places. This study is dedicated to improving the efficiency of path planning for disinfecting robots, and the APF-GFARRT* algorithm is proposed by improving the RRT* algorithm, which integrates the APF, fuzzy control, and the concept of restricted sampling domain. The superiority of the algorithm is mainly reflected in the following aspects.

The APF-GFARRT* algorithm performs well in dense environments, with an average feasible path search time of only 3.5721 s, which is much better than both RRT and RRT*, and in comparison, it saves nearly 48% of the time compared to RRT and 47% of the time resources compared to RRT*. Comparison with the original RRT and RRT* shows that the average path cost of APF-GFARRT* is decreased by 20% and 10%, respectively. In addition, the APF-GFARRT* algorithm is also able to significantly eliminate redundant nodes in the path by about 64% compared to RRT and about 37% for RRT*.

The above study confirms that APF-GFARRT* is significantly more effective in finding paths in dense environments, and is able to quickly find feasible paths with lower final path costs and fewer nodes. Due to time and capacity constraints, this simulation experiment was only completed in MATLAB. In the future, to better test the algorithm and its performance, it should be further investigated and deployed in a natural disinfection robot system for validation.

## Figures and Tables

**Table 1:** Fuzzy control rules of AdaptiveStep.

ObsDensity	PointDist				
	NL	NS	ZE	PS	PL
NL	ZE	PS	PS	PL	PL
NM	NS	ZE	PS	PS	PL
ZE	NS	NS	ZE	PS	PS
PM	NL	NS	NS	ZE	PS
PL	NL	NL	NS	NS	ZE

**Table 2:** Comparison of algorithms under Map 1.

Algorithm Name	Avg Path Nodes	Avg Path Cost	Search Success Rate
RRT	63.4727	159.0696	75.48%
RRT*	58.3754	145.8456	71.67%
APF-RRT*	57.2992	143.2481	86.55%
APF-GFARRT*	26.7362	121.0377	90.21%

**Table 3:** Comparison of algorithms in the same obstacle environment.

Algorithm Name	Avg Path Nodes	Avg Path Cost	Search Success Rate
RRT	69.4068	187.3988	46.25%
RRT*	64.8138	174.5346	47.23%
APF-RRT*	56.3792	152.3845	65.92%
APF-GFARRT*	28.2977	128.4178	85.76%

**Table 4:** Algorithm comparison.

Algorithm Name	Avg 1st Path Time/s	Avg Path Nodes	Avg Path Cost	Search Success Rate
RRT	6.8701	81.1864	160.6656	73.48%
RRT*	6.6804	46.3722	142.4439	84.98%
APF-RRT*	4.74	45.8793	138.9890	87.16%
APF-GFARRT*	3.5721	29.2977	128.184	97.62%

## Data Availability

Data are contained within the article.
